# Comparative Genomics of 12 Strains of *Erwinia amylovora* Identifies a Pan-Genome with a Large Conserved Core

**DOI:** 10.1371/journal.pone.0055644

**Published:** 2013-02-07

**Authors:** Rachel A. Mann, Theo H. M. Smits, Andreas Bühlmann, Jochen Blom, Alexander Goesmann, Jürg E. Frey, Kim M. Plummer, Steven V. Beer, Joanne Luck, Brion Duffy, Brendan Rodoni

**Affiliations:** 1 Cooperative Research Centre for National Plant Biosecurity, Bruce, Australian Capital Territory, Australia; 2 Department of Botany, La Trobe University, Bundoora, Victoria, Australia; 3 Department of Plant Pathology and Plant-Microbe Biology, Cornell University, Ithaca, New York, United States of America; 4 Biosciences Research Division, Department of Primary Industries, Knoxfield, Victoria, Australia; 5 Agroscope Changins-Wädenswil ACW, Swiss National Competence Center for Fire Blight, Plant Protection Division, Wädenswil, Switzerland; 6 CeBiTec, University of Bielefeld, Bielefeld, Germany; Universidad Pública de Navarra, Spain

## Abstract

The plant pathogen *Erwinia amylovora* can be divided into two host-specific groupings; strains infecting a broad range of hosts within the *Rosaceae* subfamily Spiraeoideae (e.g., *Malus*, *Pyrus*, *Crataegus*, *Sorbus*) and strains infecting *Rubus* (raspberries and blackberries). Comparative genomic analysis of 12 strains representing distinct populations (e.g., geographic, temporal, host origin) of *E. amylovora* was used to describe the pan-genome of this major pathogen. The pan-genome contains 5751 coding sequences and is highly conserved relative to other phytopathogenic bacteria comprising on average 89% conserved, core genes. The chromosomes of Spiraeoideae-infecting strains were highly homogeneous, while greater genetic diversity was observed between Spiraeoideae- and *Rubus*-infecting strains (and among individual *Rubus*-infecting strains), the majority of which was attributed to variable genomic islands. Based on genomic distance scores and phylogenetic analysis, the *Rubus*-infecting strain ATCC BAA-2158 was genetically more closely related to the Spiraeoideae-infecting strains of *E. amylovora* than it was to the other *Rubus*-infecting strains. Analysis of the accessory genomes of Spiraeoideae- and *Rubus*-infecting strains has identified putative host-specific determinants including variation in the effector protein HopX1_Ea_ and a putative secondary metabolite pathway only present in *Rubus*-infecting strains.

## Introduction

*Erwinia amylovora*, the causal agent of fire blight, is a destructive bacterial phytopathogen reported to occur across North America, New Zealand, Europe and the Middle East [1]. Commonly, strains of *E. amylovora* infect a broad range of host plants in the sub-family Spiraeoideae including apple, pear, cotoneaster, hawthorn and quince. However, a less prevalent group of strains has also been reported in the United States of America that infect plants in the genus *Rubus,* including blackberry and raspberry.

The Spiraeoideae-infecting strains of *E. amylovora* are thought to be relatively homogenous both genetically [2], [3] and phenotypically [4], [5] with only minor variations evident. Genetic variation has been identified in populations of Spiraeoideae-infecting *E. amylovora* using a variety of molecular fingerprinting techniques including PCR-ribotyping, pulse field gel electrophoresis (PFGE) after *Xba*I restriction, minisatellite-primed PCR, random amplified polymorphic DNA (RAPD) analysis, amplified fragment length polymorphism (ALFP) and clustered regularly interspaced short palindromic repeat (CRISPR) analysis [4], [6], [7], [8], [9], [10]. *Rubus*-infecting strains of *E. amylovora* contain greater genetic diversity than the Spiraeoideae-infecting strains [3], [8], [11]. *Rubus*-infecting strains are not pathogenic to apple [12], [13] but variation has been observed in their ability to infect immature pear fruit, with some strains being weakly virulent (causing necrosis with limited ooze production) and others unable to cause any symptoms [14]. Phenotypic differences that have been identified between the Spiraeoideae- and *Rubus*-infecting strains include variation in exopolysaccharide composition [15], carbon utilization and secreted protein profiles [13], [16], but to date, only the effector protein Eop1 has been shown to be directly involved in host specificity in *E. amylovora* [17].

The diversity of a species can be defined by analyzing the repertoire of genes represented across all strains of the species, its pan-genome. The pan-genome includes the ‘core genome’ of genes common to all strains of the species and the ‘dispensable or accessory genome’, which consists of genes present in at least one, but not all strains of a species [18]. The essence of a species, in terms of its fundamental biological processes and derived traits from a common ancestor, is linked to the core genome. However, genetic traits linked to variation in virulence, adaptation and antibiotic resistance are more often governed by the dispensable or accessory genome [19]. Pan-genome analyses of bacterial species (e.g., *Haemophilus influenzae, Escherichia coli*) have clearly shown that the genome sequence of one or two genomes per species is not sufficient to understand within-species diversity and that sequencing of multiple strains is required to present a more consistent definition of the species itself [18], [19].

To date, the genomes of two Spiraeoideae-infecting strains of *E. amylovora* [2] and the genome of one *Rubus*-infecting strain [20] have been published. Comparison of the two Spiraeoideae-infecting strains revealed them to be almost identical (99.99%) with the major differences being a large rearrangement in the chromosomal DNA and plasmid content [2]. Genome comparison of *Rubus*-infecting strain ATCC BAA-2158 to Spiraeoideae-infecting strain CFBP 1430 identified 90% of the coding sequences (CDS) to be conserved between both strains and identified 373 CDS of the ATCC BAA-2158 genome to be non-conserved (singletons) [20]. Here, the diversity of *E. amylovora* is further investigated by defining the pathogen’s pan-genome using genomes from twelve strains that were carefully selected to represent the broadest diversity, based on differential geographical origin, isolation year or PFGE patterns [8], [21], [22].

## Results and Discussion

### The Pan-genome of *E. amylovora*

**Figure 1:**
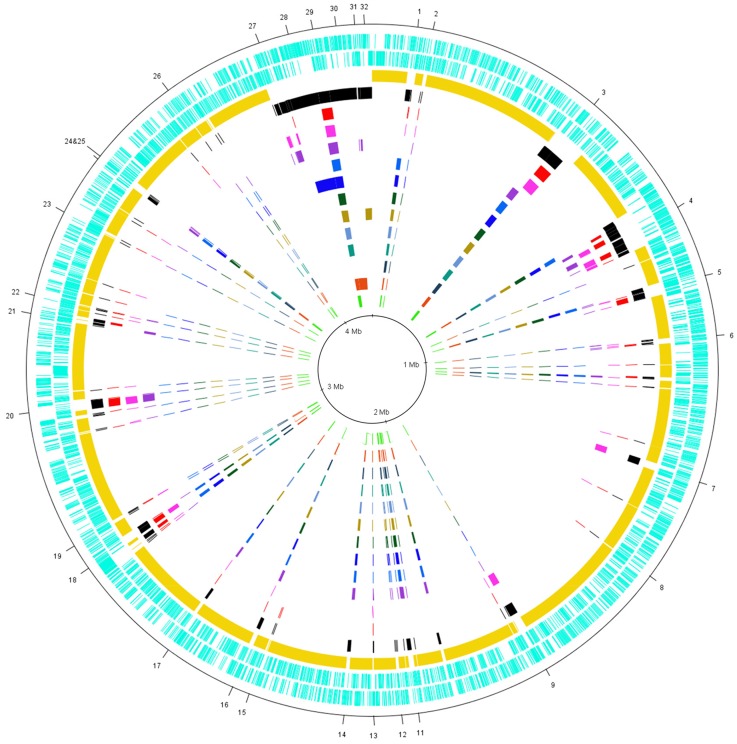
Circular plot of the pan-genome of *E. amylovora.* The CDS of the pan-genome (forward and reverse) are depicted in the two outermost circles (aqua). Moving inwards, the core genome is depicted in yellow and the accessory genome in black. The accessory genome of the individual strains of *E. amylovora* continue inwards as follows: *Rubus*-infecting strains MR1 (red), Ea644 (pink) and ATCC BAA-2158 (purple), and Spiraeoideae-infecting strains CFBP 1430 (light blue), ATCC 49946 (royal blue), Ea266 (dark green), CFBP 2585 (tan), 01SFR-BO (sky blue), Ea356 (teal), UPN527 (navy blue), ACW 56400 (orange) and CFBP 1232^T^ (light green). Variable regions of interest are numbered with a pan-genome locus (PL) of 1 to 32 and are described in Supplementary Tables 1 and 2. Of note are PL 4 (ICE flanking PAI-1), PL 20 (secondary metabolite cluster only found in *Rubus*-infecting strains), PL 27 (sequence from the *Rubus*-infecting strains that could not be assembled into contiguous sequence), PL 28 (pEA72), PL 29 (pEA29), PL 30 (pEI70), PL 31 (pEAR5.2 and pEAR4.3) and PL 32 (pEA30).

**Figure 2:**
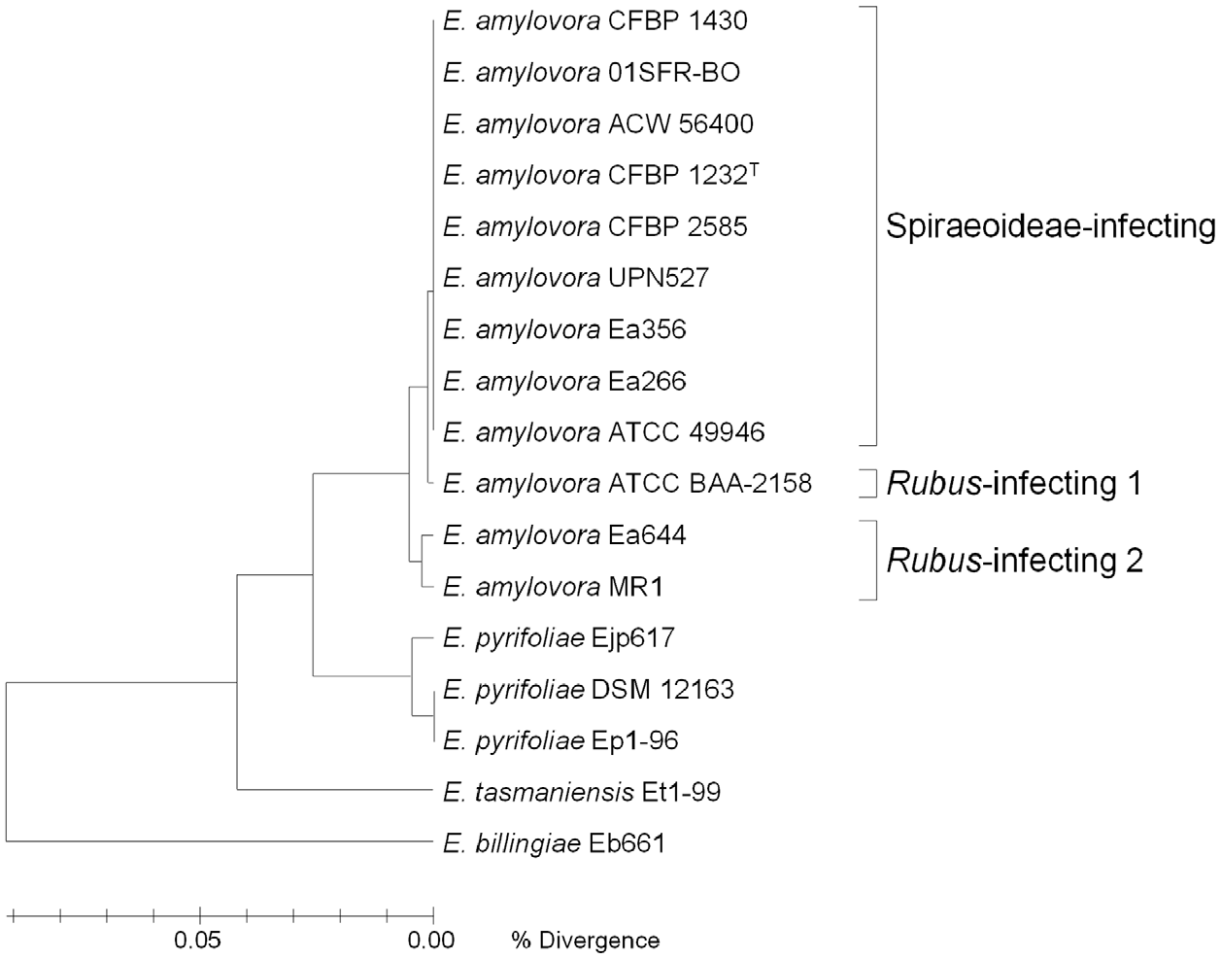
Phylogenetic analysis of *Erwinia* species created in EDGAR based on concatenated sequence of the core genome. All strains of *E. amylovora* cluster together and are separate from the other *Erwinia* species. The Spiraeoideae-infecting strains form a distinct cluster within *E. amylovora* and the *Rubus*-infecting strain ATCC BAA-2158 (*Rubus*-infecting 1) clusters more closely with these strains than with the other two *Rubus*-infecting strains labeled *Rubus*-infecting 2.

The chromosomes of the twelve genomes of *E. amylovora* compared in this study are all approximately 3.8 Mb. The Spiraeoideae-infecting strains and ATCC BAA-2158 have an average G+C content of 53.6% and the *Rubus*-infecting strains Ea644 and MR1 have G+C contents of 53.3 and 53.4, respectively (**Table 1**). Analysis of the annotated sequences revealed that 86% of the average *E. amylovora* genome consists of CDS and has an average CDS density of approximately 1 per kb. The pan-genome of *E. amylovora* was calculated to contain 5751 CDS of which 3414 CDS were considered as core (**Figure 1**). The average number of CDS predicted per genome was 3819 CDS meaning that on average 89% of each individual genome is core, though this percentage did vary between 83% (MR1) and 92% (ATCC 49946) (**Table 1**). Comparison of average amino acid identities (AAI) calculated from the core genome indicated that the core genome of *E. amylovora* is highly conserved (>99% amino acid identity among all strains) (**Table 2**). AAI and phylogenetic analysis of the core genome of *E. amylovora* strains (complete and draft) indicated that they are all part of the same species, with the Spiraeoideae-infecting strains exhibiting much less diversity than the *Rubus*-infecting strains (**Table 2** **and** **Figure 2**). The *Rubus*-infecting strains Ea644 and MR1 cluster together but the *Rubus*-infecting strain ATCC BAA-2158 clusters more closely with the Spiraeoideae-infecting strains than it does with the other *Rubus*-infecting strains. This grouping is consistent with previous studies using rep-PCR, carbon utilization and phylogeny based on *rpoB* [8], [11], [16].

**Table 1:** Strain metadata and genome sequence statistics for the 12 *E. amylovora* strains analyzed in this study.

									Chromosome metrics					
Strain name (synonyms)	Isolated from	Origin	Genome status	Replicon	Accession numbers			Length (bp)	G+C content	Number of CDS	% coding	CD density (per kb)		Reference
Spiraeoideae-infecting isolates														
CFBP 1430	*Crataegus*	France, 1972	Complete	Chromosome	FN434113			3805573	53.6	3706	85.6	0.973		[2], [61]
				pEA29	FN434114			28259						
ATCC 49946 (Ea273)	*Malus domestica*	New York, USA, 1973	Complete	Chromosome	FN666575			3805874	53.6	3712	85.1	0.903		[2]
				pEA72	FN666577			71487						
				pEA29	FN666576			28243						
CFBP 1232^T^ (NCPPB 683^T^)	*Pyrus communis*	UK, 1959	Draft	Chromosome	CAPB01000001-42			3767276	53.6	3780	86.6	1.003		[62]
				pEA29	HF560650			28251						
CFBP 2585 (Ea495)	*Sorbus* sp.	Ireland, 1986	Draft	Chromosome	CAOZ01000001-12			3767556	53.6	3734	86.0	0.992		[63]
				pEA30	HF560646			29586						
				pEA29	HF560645			28258						
Ea266 (E4001A)	*Malus* sp.	Ontario, Canada	Draft	Chromosome	CAOY01000001-38			3758663	53.6	3804	86.1	1.011		[22]
				pEA29	HF560644			28261						
Ea356 (Ea1/79)	*Cotoneaster* sp.	N-Germany, 1979	Draft	Chromosome	CAOX01000001-14			3763948	53.6	3760	86.2	0.998		[22]
				pEA29	HF560643			28258						
01SFR-BO	*Sorbus* sp.	Ravenna, Italy, 1991	Draft	Chromosome	CAPA01000001-11			3767556	53.6	3744	86.1	0.993		[7]
				pEA29	HF560647			28259						
ACW 56400	*Pyrus communis*	Fribourg, Switzerland, 2007	Draft	Chromosome	AFHN01000001-22			3766903	53.6	3758	86.5	0.995		[6]
				pEI70	CP002951			65831						
				pEA29	AFHN01000023			28251						
UPN527	*Malus* sp.	Navarra, Spain, 1997	Draft	Chromosome	CAPC01000001-18			3766971	53.6	3746	86.1	0.994		[7]
*Rubus*-infecting isolates														
ATCC BAA-2158 (BB1, IL5, Ea246, BC204)	*Rubus* sp.	Illinois, USA, 1972	Draft	Chromosome	FR719181-209			3808219	53.6	3827	86.2	1.004		[12], [20]
				pEA29	FR719212			28138						
				pEAR5.2	FR719211			5251						
				pEAR4.3	FR719210			4369						
Ea644	*Rubus idaeus* cv. Polana	Massachusetts, USA, 2003	Draft	Chromosome	CAPD01000001-40		3803638		53.3	3937	86.4	1.034		[11]
				pEA29	HF560648		28689							
MR1 (Ea574)	*Rubus idaeus*	Michigan, USA	Draft	Chromosome		CAPE01000001-29	3789707		53.4	4042	84.7	1.067		[8]
				pEA29		HF560649	27604							

**Table 2:** Percent average amino acid identities (AAI) calculated from the core genome data set using EDGAR and MUMi scores of genomic distance between the 12 *E. amylovora* strains and closely related *Erwinia* spp.

	Average amino acid identities (%)			MUMi Scores		
Data set	Average (Stdev)	Min	Max	Average (Stdev)	Min	Max
All *E. amylovora*	99.72 (0.34)	99.19	100	0.044 (0.050)	0.000	0.122
All Spiraeoideae-infecting isolates	99.98 (0.02)	99.93	100	0.005 (0.003)	0.000	0.010
All *Rubus*-infecting isolates	99.42 (0.36)	99.19	99.83	0.089 (0.050)	0.031	0.119
ATCC BAA-2158 to all Spiraeoideae-infecting isolates	99.79 (0.02)	–	–	0.044 (0.002)	–	–
ATCC BAA-2158 to Ea644 and MR1	99.23/99.19	–	–	0.116/0.118	–	–
MR1 to all Spiraeoideae-infecting isolates	99.21 (0.00)	–	–	0.118 (0.002)	–	–
Ea644 to all Spiraeoideae-infecting isolates	99.24 (0.02)	–	–	0.116 (0.002)	–	–
MR1 to Ea644	99.83	–	–	0.031	–	–
*E. amylovora* to *E. pyrifoliae*	95.44 (0.05)	–	–	0.588 (0.004)	–	–
*E. amylovora* to *E. tasmaniensis* Et1/99	92.66 (0.01)	–	–	0.804 (0.001)	–	–
*E. amylovora* to *E. billingiae* EB661	85.07 (0.01)	–	–	0.940 (0.000)	–	–

MUMi score values vary from 0 for identical genomes to 1 for very distant genomes.

We performed maximal unique matches index (MUMi) analysis to determine intra-species and intra-genus whole genome diversity of each genome analyzed in this study and with closely related species *E. pyrifoliae*, *E. tasmaniensis* and *E. billingiae* (**Table 2**). MUMi scores of genomic distance ranging from 0 to 1 correlate with average nucleotide identity scores and multi locus sequence typing with a score of 0 for identical genomes to 1 for very distant genomes [23]. MUMi scores of *E. amylovora* genomes complemented phylogenetic analysis showing significant similarity among all *E. amylovora* strains (0.000–0.122) compared with closely related species (0.585–0.941), and in particular, high homogeneity among Spiraeoideae-infecting strains (0.000–0.008). MUMi scores also indicate that ATCC BAA-2158 is more closely related to Spiraeoideae-infecting strains (0.043–0.047) than the other *Rubus*-infecting strains (0.116–0.119). MUMi scores show that *Rubus*-infecting strains Ea644 and MR1 are most genetically similar to each other (0.031) and are as genetically similar to ATCC BAA-2158 as they are to the Spiraeoideae-infecting strains (0.114–0.122), corresponding to AAI analysis (**Table 2**) and phylogenetic analysis (**Figure 2**).

In comparison with other microbial pan-genome studies, *E. amylovora* has a high percentage of CDS per individual genome classified as core (**Table 3**). This highlights the relatively small amount of intra-species genetic diversity observed in *E. amylovora* even with the inclusion of the more genetically diverse *Rubus*-infecting strains. It has been speculated that *E. amylovora* has relatively low genetic diversity (compared to other plant pathogens like *P. syringae*) because it undergoes limited genetic recombination, it has a high degree of specialization to a narrow ecological niche and in Spiraeoideae-infecting strains, is exposed to limited selection pressures due to pome fruit breeding strategies favoring high-value varieties, that often are highly susceptible to fire blight [8], [24].

**Table 3:** Percentage of CDS predicted to be core in the genome of each selected bacterial species.

Species	% core CDS	No. Genomes		Reference
*Escherichia coli*	44%	17	Open	[64]
*Ralstonia solanacearum*	48%	6	Unknown	[65]
*Xanthomonas oryzae*	63%	4	Open	[26]
*Pseudomonas syringae*	64%	19	Unknown	[66]
*Streptococcus pneumoniae*	74%	44	Open	[67]
*Xanthomonas campestris*	75%	5	Open	[26]
*Listeria monocytogenes*	80%	26	Open	[68]
*Staphylococcus aureus*	85–89%	17	Closed	[69]
*Erwinia amylovora*	89%	12	Open	This study
*Mycobacterium tuberculosis*	98%	9	Closed	[70]

**Figure 3:**
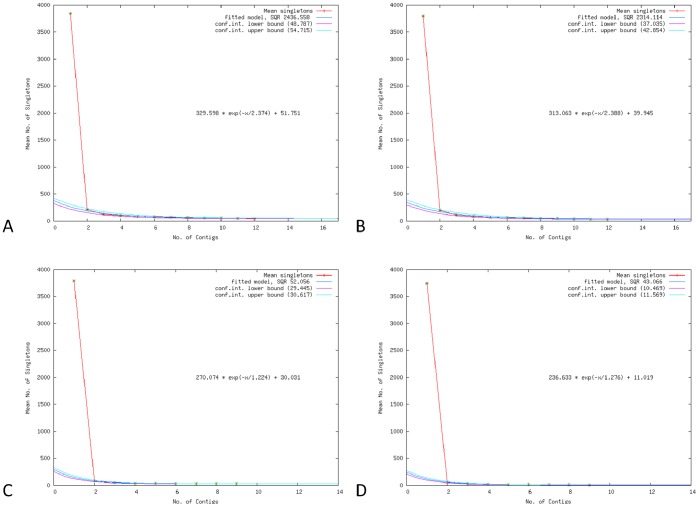
Singleton development plot analysis. Single development plots defined using 12 strains of *E. amylovora* including plasmids (A) and excluding plasmids (B), and 9 Spiraeoideae-infecting strains of *E. amylovora* including plasmids (C) and excluding plasmids (D). All plots indicate that the pan-genome of *E. amylovora* is ‘open’, predicting that each additional strain sequenced will add 52 (Plot A), 40 (Plot B), 30 (Plot C) and 11 (Plot D) new singletons to their respective pan-genome sets.

The number of genomes required to estimate the size of a species’ pan-genome has been mathematically modeled [19], [25] leading to the concept of ‘open’ and ‘closed’ pan-genomes. In an open pan-genome new genes are added to the gene repertoire of the species with every new strain sequenced [19]. Based on EDGAR analysis [26] using two complete genome sequences and ten draft genome sequences of *E. amylovora*, the pan-genome is predicted to be open (**Figure 3A**). Singleton development analysis estimated that 52 novel CDS (including plasmids) and 40 novel CDS (excluding plasmids) (**Figure 3B**) would be added to the pan-genome with each additional genome of *E. amylovora* sequenced.

### Variation among the Spiraeoideae-infecting Strains

Phylogenetic and MUMi analysis have shown that Spiraeoideae-infecting strains of *E. amylovora* are highly homogeneous at the chromosome level, which is consistent with previous studies [2]. When a singleton development analysis using only the Spiraeoideae-infecting strains with nearly identical chromosomes was conducted in EDGAR (including plasmids), the pan-genome of this subgroup was open (**Figure 3C**) with a prediction of 30 new genes to be added to the pan-genome with each additional genome sequenced. When the same analysis was done excluding plasmids the pan-genome of Spiraeoideae-infecting strains was still predicted to be open with 11 new genes to be added to the pan-genome with each additional genome sequenced (**Figure 3D**) highlighting the important role plasmids play in the genetic diversity of *E. amylovora*. It is likely that the figures for all of the pan-genome calculations are slightly inflated due to the use of draft genomes (i.e., with contig breaks that influence CDS prediction and comparison) and that the pan-genome of the Spiraeoideae-infecting strains, excluding plasmids, is closed.

Recently, Spiraeoideae-infecting strains of *E. amylovora* have also been differentiated into different geographical groups based on CRISPRs [6], [27]. CRISPR analysis clustered Spiraeoideae-infecting strains of *E. amylovora* into three main groups, two of which contained strains only from North America (CRISPR groups II & III) and one that contained strains from Europe, the Middle East, New Zealand and from the east coast of North America (CRISPR group I). The more phylogenetically distant clusters of groups I and III correlated with earlier PCR ribotyping experiments that also grouped *E. amylovora* strains into clusters of geographical origin based on genetic differences [8]. All sequenced Spiraeoideae-infecting strains analyzed in this study are of CRISPR group I [6]. Further investigation into *E. amylovora* strains of CRISPR groups II and III may identify more genetic diversity than exists among Spiraeoideae-infecting strains in this study.

### Variation among All Strains of *E. amylovora* – the Accessory Genome

The majority of diversity observed within the pan-genome of *E. amylovora* was between the Spiraeoideae-infecting and the *Rubus*-infecting strains and among the individual *Rubus*-infecting strains. Cross-infectivity of *Rubus*-infecting strains on Spiraeoideae and vice versa is rare [11], [13], and it is hypothesized that the genetics influencing host-specificity determination is present within the accessory genes of the pan-genome. Given the lack of diversity observed in the chromosomes of the Spiraeoideae-infecting strains we have used *E. amylovora* CFBP 1430 to represent the Spiraeoideae-infecting strains in this section although all strains were included in the analysis. Variable regions of the pan-genome (**Figure 1**) are summarized in **Supplementary Tables S1 and S2** with regions of note discussed in more depth in the following sections.

#### Genomic islands

Genomic islands (GIs) are defined as clusters of genes in prokaryotic genomes of probable horizontal origin and include prophages, integrated plasmids, integrative conjugative elements, integrons and conjugative transposons [28]. GIs typically encode mobility related genes but also carry significant “cargo” genes that can be involved in virulence, drug resistance and increased ecological fitness [29], [30], [31]. We have identified 12 loci within the *E. amylovora* pan-genome which vary in GI content among strains (**Supplementary Table S1 and** **Figure 1**) and which account for a large proportion of the genetic variation observed within the chromosomal component of the pan-genome. The majority of CDS identified on GI’s of the *E. amylovora* pan-genome encode hypothetical proteins and mobility related genes (**Supplementary Table S1**), including genes involved in replication, transfer and integration of mobile elements.

The largest GI in any of the *E. amylovora* strains (34.5 kb) is present in the *Rubus*-infecting strains Ea644 and MR1 at pan-genome locus (PL) 3 (**Figure 1**). At the same locus in the Spiraeoideae-infecting strains and ATCC BAA-2158, there is a different GI of approximately 23.4 kb. Analysis of the CDS predicted across these GIs indicates that both GIs at this locus carry different types of bacterial host-specific modification systems responsible for protecting the cell from foreign DNA. These modification systems generally have two primary functions; protection of host DNA (bacterial) and degradation of foreign DNA with restriction enzymes [32]. Ea644 and MR1 encode a type 1 restriction modification system, a system which protects the host DNA by adding methyl groups to recognition sites of expressed restriction enzymes [32] and the Spiraeoideae-infecting strains encode a DNA degradation (Dnd) host-specific modification system which (in other bacteria) incorporates sulfur into the DNA backbone to prevent restriction recognition [33].

Only one GI of approximately 20 kb (**Figure 1** - PL20) was present in all of the *Rubus*-infecting strains of *E. amylovora* but absent in Spiraeoideae-infecting strains. Remnants of PL20 were found in CRISPR region 1 (CRR1) of the Spiraeoideae-infecting strains, suggesting that this island in *Rubus*-infecting strains is ancestral to CRR1 of the Spiraeoideae-infecting strains [6]. PL20 encodes three polyketide synthase proteins (EAIL5_2889, EAIL5_2891 and EAIL5_2892), a non-ribosomal peptide synthase (EAIL5_2890) alongside a putative transporter (EAIL5_2885) (**Supplementary Figure S1**). Other genes in this cluster are modifying enzymes. As the total gene cluster represents a novel NRPS/PKS, the prediction of the final chemical structure of the product is impossible.

#### Pathogenicity and host specificity determinants

Two major virulence determinants required for *E. amylovora* to infect and cause disease on host plants are the exopolysaccharide amylovoran biosynthesis pathway and the Hrp type III secretion system (T3SS). There are no major differences among the 12 strains of *E. amylovora* in the amylovoran biosynthesis cluster (>98% amino acid identity across the whole region) or the Rcs phosphorelay system that controls its regulation [34]. There is however, variation within Hrp cluster of *E. amylovora* (**Figure 1** - PL4) [35]. The Hrp cluster is a pathogenicity island that encodes the hypersensitive response and pathogenicity (hrp) T3SS and the majority of the known T3SS effector proteins [36]. Variation was identified in HrpK (truncated in ATCC BAA-2158), the putative chaperones OrfA and OrfC (which varied between host specific groupings of *Rubus*- and Spiraeoideae-infecting strains) and more significantly, Eop1 which has been shown to function as a host limiting factor [17], [35].

The remnants of an integrative conjugative element (ICE) (previously referred to as the IT region) were present at the flank of the Hrp cluster, which differs between Spiraeoideae- and *Rubus*-infecting strains, as well as among the individual *Rubus*-infecting strains [35]. This remnant ICE is mosaic in nature with varying ICE-related genes identified in all strains, however, it appears to have undergone significant genome reduction in the Spiraeoideae-infecting strains, being more than 30 kb shorter in length than all of the *Rubus*-infecting strains sequenced thus far [35].

Additional T3SS effector proteins that are located outside the Hrp T3SS cluster in the *E. amylovora* genome have also been identified and include: AvrRpt2_Ea_ (Eop4) a protein found to contribute to virulence on immature pear fruit [37]; HopPtoC an effector protein induced during infection on immature pear fruit [38]; HopAK1_Ea_ (Eop2) a predicted translocator; and HopX1_Ea_ (Eop3) a protein conditioning avirulence on apple [39]. Comparison of effector homologues in the pan-genome found HopPtoC and HopAK1_Ea_ are present in all strains of *E. amylovora* (≥95% amino acid identity). However, analysis revealed variation of the effector proteins HopX1_Ea_ and AvrRpt2_Ea_ among different strains of *E. amylovora*. Comparison of the region encoding HopX1_Ea_ identified that the *Rubus*-infecting strains only contained sequence encoding the last 72–85 amino acids of the C-terminal end of Spiraeoideae-infecting HopX1_Ea_. A recent study hypothesized that the 301 residue protein HopX1_Ea273_ is recognized by the host plant [39] so the consistent variation observed here among Spiraeoideae-infecting and *Rubus*-infecting strains of *E. amylovora* make this protein a strong candidate as a host specificity determinant. A single base deletion at nucleotide 165 (amino acid 55) of AvrRpt2_Ea_ in *Rubus*-infecting strains Ea644 and MR1, has caused a frameshift resulting in a truncation at amino acid 73. Annotation of this region in Ea644 and MR1 predicts a CDS for AvrRpt2 which correlates with amino acids 79 to 223 of AvrRpt2 of the Spiraeoideae-infecting strain CFBP1430. The lack of an N-terminal signal, which is important for secretion, translocation, and chaperone binding of other T3SS effector proteins [40], in either of these T3SS effector proteins may result in an inability to be translocated into the host cell.

#### Type VI secretion systems

Type VI secretion systems (T6SS) have been identified in at least a quarter of the sequenced Gram-negative bacteria [41]. Three T6SS gene clusters have been identified in *E. amylovora* [2] but their exact role in this species is unknown. Inter-species comparison of the T6SS clusters among closely related *Erwinia* and *Pantoea* species has previously identified conserved core regions and variable *hcp* and *vgrG* islands [42].

Pan-genome comparison has shown that there is no variation among the Spiraeoideae-infecting isolates, but has identified variation between Spiraeoideae-infecting isolates and the *Rubus*-infecting strains and among the *Rubus*-infecting isolates in the T6SS clusters 1 and 3 (detailed in the **Supplementary Text** and **Supplementary Figures S2 and S3**).

Within the conserved core regions of the three T6SS, variation was observed within the region III of T6SS-1. This variation included *Rubus*-infecting strains Ea644 and MR1 each containing additional sequence (approximately 1300 bp sharing 99% identity) between COG3520 and *clpV* (**Supplementary Figure S2**), encoding proteins with sequence identity (52–65% aa identity) to CDS in the corresponding loci of the T6SS-1 of *E. pyrifoliae* DSM 12163 (EPYR_00667 and EPYR_00668) [42], [43].

Variation between strains of *E. amylovora* was primarily found within the non-conserved *hcp* and *vgrG* islands of T6SS-1 regions II and IV and T6SS-3 region IV. These variable regions share high sequence similarity to closely related bacteria of the genera *Erwinia* and *Pantoea*. The identification of intra-species diversity in the *hcp* and *vgrG* islands of *E. amylovora* confirm that these regions are hot-spots for rearrangement and are likely to play an important role in the evolution and functional diversification of T6SS [42].

#### Carbohydrate utilization

*E. amylovora* CFBP 1430 is able to utilize L-arabinose as a carbon source using the proteins encoded by the *araABFGHC* gene cluster (EAMY_1725–1730), which convert L-arabinose to D-xylulose 5-phospate for downstream purposes [44]. Unlike in all of the Spiraeoideae-infecting strains and ATCC BAA-2158 (**Figure 1** - PL10), the *Rubus*-infecting strains MR1 and Ea644 both lack the sequence corresponding to gene cluster containing *araABFGH*, but the regulatory gene *araC* (BN439_2117 and BN440_2152) is present. Though it will need to be functionally confirmed, these findings indicate an inability of the *Rubus*-infecting strains MR1 and Ea644 to metabolize and actively transport L-arabinose.

Another region of variation in the pan-genome of *E. amylovora* that appears to have metabolic implications is PL11, which is found in Spiraeoideae-infecting strains and ATCC BAA-2158. This 11.3 kb region contains CDS encoding proteins commonly involved in carbon utilization and transport, including multiple monooxygenase domain encoding CDS, an acyl-CoA dehydrogenase, a peptidase and putative sugar transport protein. Based on the annotation of these CDS, it is difficult to predict a substrate for this cluster.

#### Plasmids

Plasmids are a primary source of genetic diversity among *E. amylovora* strains, particularly in the Spiraeoideae-infecting strains. We sequenced six plasmids comprising 4.7% of the pan-genome of *E. amylovora* but found only five of the fourteen currently known plasmids [45] within our 12 genomes. The nearly ubiquitous and diagnostic plasmid pEA29 (**Figure 1**– PL29) which encodes genes for thiamine biosynthesis [46] was present in all strains except for UPN527 (**Table 1**). Loss of the plasmidic *thiOSGF* thiamine biosynthetic genes, results in thiamine prototrophy [47]. However, the strain UPN527 is still virulent, indicating that thiamine prototrophy can be overcome in the host.

Plasmid pEA72 (**Figure 1** - PL28), which has functionally annotated CDS including a type IV secretion system, potentially involved in conjugative transfer of the plasmid [48], but has no known function to date, was only present in strain ATCC 49946 (**Figure 1**). In ATCC BAA-2158 we found two small circular plasmids pEAR5.2 and pEAR4.3 of unknown function (**Figure 1** - PL31) [20]. In a previous study, three small plasmids were identified in ATCC BAA-2158 [49] but the third, pEA2.8, which contains a CDS for the ampicillin resistance protein beta-lactamase (though this has not been functionally explored), appears to have been lost by this isolate of ATCC BAA-2158. However, we have confidence that the phenotypic information presented for this strain is correct as other studies conducted in the laboratory with the same strain of ATCC BAA-2158 included phenotypic analysis [17], [20].

The genome sequence of strain CFBP 2585 revealed a novel *E. amylovora* plasmid pEA30 of approximately 30 kb (**Figure 1**). This plasmid contains a type IV secretion system for putative conjugative plasmid transfer and predicted CDS involved in plasmid replication and maintenance (**Supplementary Figure S4**). Nucleotide similarity searches to known sequences in GenBank indicate that pEA30 is most closely related to the RA3 plasmid of *Aeromonas hydrophila* (i.e., 70% total sequence coverage and 64–81% identity of all high-scoring segment pair matches). The RA3 plasmid is the archetype of the IncU plasmids, which are a distinct group of mobile elements with highly conserved backbone functions and variable antibiotic resistance gene cassettes [50]. Similarity between pEA30 and RA3 is limited to the conserved backbone of replication, maintenance and transfer-related genes (**Supplementary Figure S4**) and pEA30 does not contain any known antibiotic resistance cassettes, leaving the function of this plasmid, like many of the other *E. amylovora* plasmids [48], cryptic.

The genome sequence of strain ACW 56400 from Switzerland contained the recently described plasmid pEI70, which contains an ICE as a major feature and has thus far only been reported in European *E. amylovora* populations [45]. The precise function of pEI70, which has high sequence similarity to pEB102 from the epiphyte *E. billingiae* Eb661, is to a large extent unknown and it is thought that the ICE is unable to integrate into the chromosome of *E. amylovora* [45]. However, it has been demonstrated that this plasmid has an effect on strain aggressiveness in immature pear fruit assays and, given its similarity to pEB102, it is postulated that pEI70 may improve environmental fitness of the possessing strain *in planta* rather than contributing directly to enhanced virulence [45].

### Conclusion

Individual genomes of the *E. amylovora* are largely made up of core CDS, with approximately 10% being variable among strains. “Mining” the accessory genomes of the *Rubus*-infecting strains has identified additional clues to the possible mechanisms influencing host-specificity in *E. amylovora*. All *Rubus*-infecting strains analyzed in this study possess a putative secondary metabolite pathway and a multi-gene substitution in the LPS biosynthesis pathway [11] not found in Spiraeoideae-infecting strains. Variation was also observed in effector proteins of *Rubus*-infecting strains including the host limiting factor Eop1 (as previously described [17]) and the avirulence protein HopX1_Ea_. There was significant difference between the HopX1_Ea_ of *Rubus*- and Spiraeoideae-infecting strains, with *Rubus*-infecting strains missing the coding sequence for more than two thirds of the Spiraeoideae type HopX1_Ea_ at the N-terminus of the protein.

Overall, more genetic variation was observed among the *Rubus*-infecting strains of *E. amylovora* compared to the Spiraeoideae-infecting strains. As has been described previously [8], [11], we found that ATCC BAA-2158 was genetically more similar to the Spiraeoideae-infecting strains than to the other *Rubus*-infecting strains. Previously, when carbon utilization analysis was used to differentiated *Rubus*-infecting strains into different groups, one group was identified as being more Spiraeoideae-like [16]. The identification of clusters of genes involved in carbon utilization present in the Spiraeoideae-infecting strains and ATCC BAA-2158 in this study provides support for those findings. The availability of three genomes of *Rubus*-infecting *E. amylovora* strains will aid in the facilitation of research into understanding the differences between Spiraeoideae-infecting and *Rubus*-infecting strains.

Outside the addition of plasmids, no variation was apparent in the genetic content of the Spiraeoideae-infecting strains in this study. However, Spiraeoideae-infecting strains with identical plasmid content (e.g. only pEA29) do not always exhibit identical phenotypes [22], [51]. Differential gene expression has been identified as a cause for varied virulence phenotypes in Spiraeoideae-infecting strains of *E. amylovora* [51] but the underlying genetic cause for this variation is unknown. Exploration of the transcriptome and the metabolome of Spiraeoideae-infecting strains (and *Rubus*-infecting strains) of *E. amylovora* would certainly aid in identifying factors contributing to phenotypic diversity.

Defining the pan-genome of *E. amylovora* has allowed us to gain a better understanding of the species as a whole. Compared with other bacterial species, *E. amylovora* does not possess a great deal of genetic diversity. Understanding how this limited genetic diversity contributes to different phenotypes will eventually pave the way for improved diagnostics and, ultimately, better control strategies for this destructive pathogen.

## Methods

### Strain Selection

Based on the host and year of isolation, worldwide geographic origin and the PFGE patterns, we selected a total of nine diverse strains of *E. amylovora* representing isolates from two continents, seven host plants and a time span of five decades (**Table 1**) for draft genome sequencing [22]. The complete genomes of CFBP 1430 and ATCC 49946 [2] and the draft genome of ATCC BAA-2158 [20] were also used in this analysis.

### DNA Extraction

Genomic DNA for Ea356, Ea266, CFBP 2585, MR1 and Ea644 was isolated at Cornell University using the Qiagen Blood and Cell Culture DNA Midi Kit (#13343) and for strains ACW 56400, 01SFR-BO, CFBP 1232^T^ and UPN527 at ACW using the Wizard Genomic DNA Purification Kit (Promega, Madison, WI, USA).

### Sequencing

Whole-genome sequencing of *E. amylovora* strains Ea356, CFBP 2585 and Ea266 was performed at the Victorian AgriBiosciences Research Centre, Australia using a 454 FLX pyrosequencer (454 Life Sciences, Roche, Branford, CT, USA) according to manufacturer’s instructions. Strains MR1 and Ea644 were sequenced at ACW, Switzerland using a 454 GS-Junior sequencer according to the manufacturer’s instructions. Strains ACW5 6400, 01SFR-BO, CFBP 1232^T^, UPN527 were sequenced by GATC using 36-base paired-end sequencing on an Illumina Genome Analyzer. Results of the sequencing are shown in **Supplemental Table S3**.

### Assembly and Annotation

Genomic data were assembled using Newbler (454 Life Sciences), *in silico* gap closure was performed with Lasergene (DNASTAR, Madison, WI, USA) and final assemblies were confirmed by realigning reads against the consensus assembly using NGen 2.0 (DNASTAR). All plasmid sequences reported in this study were completely assembled and circular, and chromosomal sequences were assembled to the “high quality draft sequence” level. Gaps within the sequences were mainly found in repetitive elements, e.g., the seven rRNA operons or *rhs* genes.

Genes were predicted using a combined strategy [52] based on the gene prediction programs Glimmer [53] and Critica [54]. Subsequently, the potential function of each predicted gene was automatically assigned using the GenDB annotation pipeline [55]. The resulting genome annotation was curated manually, and metabolic pathways were identified using the KEGG pathways [56] tool in GenDB.

### Genome Comparisons

The program EDGAR [26] was used to compare (predicted) protein repertoires of all strains and calculate the pan-genome, singleton and core CDS numbers. EDGAR was also used to generate the whole genome phylogenetic tree and create singleton development plots. Due to the fact that EDGAR compares predicted CDS against predicted CDS, we also used mGenomeSubstractor [57](using CFBP 1430 as the reference genome) with an h-value >0.81 cut off to eliminate annotation bias when determining the core genome. BLAST algorithms [58] were used to compare specific CDS to known sequences in GenBank.

The average amino acid identity (AAI) was calculated as described previously [59]. The maximal unique exact matches index (MUMi) distance calculation was performed using the Mummer program (version 3.20). Mummer was run on concatenated contigs (achieved by inserting a terminator string in each reading frame at each contig join) of each genome. The distance calculations performed using the MUMi algorithm are based on the number of maximal unique matches of a given minimal length shared by two genomes being compared. MUMi values vary from 0 for identical genomes to 1 for very distant genomes [23].

The program antiSMASH [60] was used for secondary metabolite gene cluster identification and core structure prediction for the putative product.

## Supporting Information

Figure S1 **Analysis of the nonribosomal peptide and polyketide biosynthesis gene cluster found only in the** ***Rubus*** **-infecting strains of** ***E. amylovora*** **(remnants of which are identified in CRISPR region 1 in the Spiraeoideae-infecting strains) using the software AntiSmash.** Using sequence from *E. amylovora* strain ATCC BAA-2158, five CDS were predicted to be part of this pathway (shaded in pink) (a) and the domains within each of the five CDS were identified (b). The domains identified include beta-ketoacyl synthase domains (green KS), phosphopantetheine attachment sites (blue PCP), AMP-binding sites (purple A), condensation domain (blue C), dehydration domain (DH), ketoreductase domains (KR) and an acyl transferase domain (AT). Additionally, the predicted core chemical structure of the product of the nonribosomal peptide or polyketide biosynthesis gene cluster is depicted (C).
(PDF)

Figure S2 **Comparison of the T6SS-1 loci from different strains of** ***E. amylovora*** **.** CDS encoding conserved core T6SS proteins are shaded in green (located in regions I and III), CDS encoding T6SS effector proteins Hcp and VrgG are colored red (located in regions II and IV, the *hcp* and *vgrG* islands), non-core CDS that are conserved among all strains are dark grey, non-conserved CDS of the T6SS that vary among strains are not colored (regions II, III and IV) and CDS flanking the T6SS are light grey. Regions of homology among strains are represented by grey shading.
(PDF)

Figure S3 **Comparison of the T6SS-3 loci from different strains of** ***E. amylovora*** **.** CDS encoding conserved core T6SS proteins are shaded in green (primarily conserved core regions I, III and V but there is also a core protein in region IV of CFBP 1430 and ATCC BAA-2158), CDS encoding T6SS effector proteins Hcp and VrgG are colored red (located in conserved core region I and *hcp* and *vgrG* islands regions II and IV), non-core CDS that are conserved among all strains are dark grey, non-conserved CDS of the T6SS are not colored (region IV) and CDS flanking the T6SS are light grey. Regions of conservation among strains are represented by grey shading.
(PDF)

Figure S4 **Comparison of plasmid pEA30 of CFBP 2585 (Ea495) to the RA3 plasmid of** ***Aeromonas hydrophila*** **.** The RA3 plasmid is the archetype of the IncU plasmids which are a distinct group of mobile elements with highly conserved backbones and variable antibiotic resistance gene cassettes. Conservation between pEA30 and RA3 (represented by the grey shaded lines) is limited to the conserved backbone of replication, maintenance and transfer related genes.
(PDF)

Table S1 **Pan-genome loci of the** ***E. amylovora*** **pan-genome that contain genomic islands.** When two lines are present for a pan-genome locus, two different genomic islands are present.
(PDF)

Table S2 **Variable regions of interest in the pan-genome of** ***E. amylovora.*** When two lines are present for a pan-genome locus, two different genomic islands are present.
(PDF)

Table S3 **Statistics for the draft assemblies of nine** ***E. amylovora*** **strains sequenced in this study.**
(PDF)

Text S1 **Additional text describing differences in lipopolysaccharides and type VI secretion systems among the** ***E. amylovora*** **genomes.**
(PDF)
