# Supplementary material for: Comparative Genomics of 12 Strains of Erwinia amylovora Identifies a Pan-Genome with a Large Conserved Core
Source: PLoS One. 2013 Feb 7;8(2):e55644. doi: 10.1371/journal.pone.0055644 (PMC3567147; doi:10.1371/journal.pone.0055644)
Supplement: Figure S1 — Analysis of the nonribosomal peptide and polyketide biosynthesis gene cluster found only in the Rubus -infecting strains of E. amylovora (remnants of which are identified in CRISPR region 1 in the Spiraeoideae-infecting strains) using the software AntiSmash. Using sequence from E. amylovora strain ATCC BAA-2158, five CDS were predicted to be part of this pathway (shaded in pink) (a) and the domains within each of the five CDS were identified (b). The domains identified include beta-ketoacyl synthase domains (green KS), phosphopantetheine attachment sites (blue PCP), AMP-binding sites (purple A), condensation domain (blue C), dehydration domain (DH), ketoreductase domains (KR) and an acyl transferase domain (AT). Additionally, the predicted core chemical structure of the product of the nonribosomal peptide or polyketide biosynthesis gene cluster is depicted (C). (PDF) [file pone.0055644.s001.pdf]

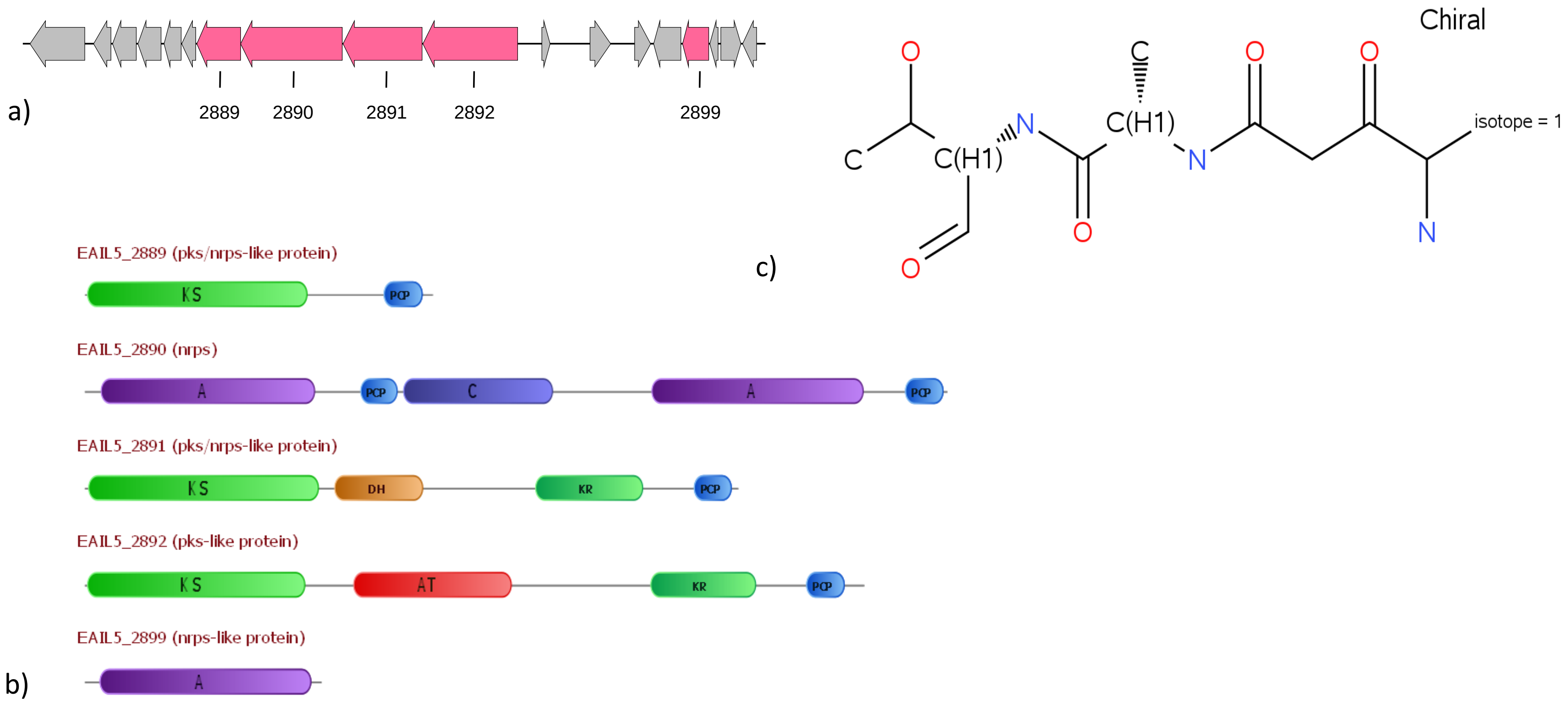

Supplementary Figure 1. Analysis of the nonribosomal peptide and polyketide biosynthesis gene cluster found only in the *Rubus*-infecting strains of *E. amylovora* (remnants of which are identified in CRISPR region 1 in the Spiraeoideae-infecting strains) using the software AntiSmash. Using sequence from *E. amylovora* strain ATCC BAA-2158, five CDS were predicted to be part of this pathway (shaded in pink) (a) and the domains within each of the five CDS were identified (b). The domains identified include beta-ketoacyl synthase domains (green KS), phosphopantetheine attachment sites (blue PCP), AMP-binding sites (purple A), condensation domain (blue C), dehydration domain (DH), ketoreductase domains (KR) and an acyl transferase domain (AT). Additionally, the predicted core chemical structure of the product of the nonribosomal peptide or polyketide biosynthesis gene cluster is depicted (C).
