# Supplementary material for: Comparative Genomics of 12 Strains of Erwinia amylovora Identifies a Pan-Genome with a Large Conserved Core
Source: PLoS One. 2013 Feb 7;8(2):e55644. doi: 10.1371/journal.pone.0055644 (PMC3567147; doi:10.1371/journal.pone.0055644)
Supplement: Figure S2 — Comparison of the T6SS-1 loci from different strains of E. amylovora . CDS encoding conserved core T6SS proteins are shaded in green (located in regions I and III), CDS encoding T6SS effector proteins Hcp and VrgG are colored red (located in regions II and IV, the hcp and vgrG islands), non-core CDS that are conserved among all strains are dark grey, non-conserved CDS of the T6SS that vary among strains are not colored (regions II, III and IV) and CDS flanking the T6SS are light grey. Regions of homology among strains are represented by grey shading. (PDF) [file pone.0055644.s002.pdf]

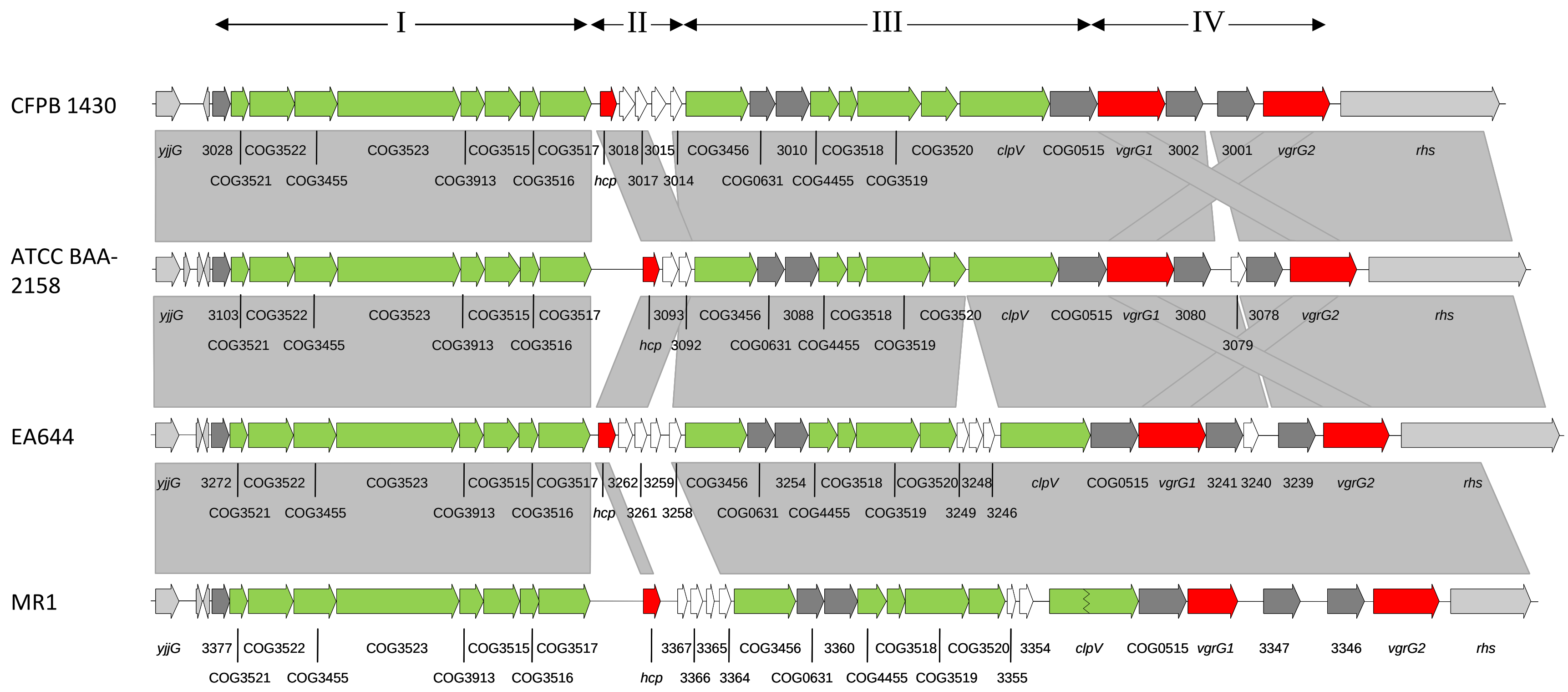

Supplementary Figure 2. Comparison of the T6SS-1 loci from different strains of *E. amylovora*. CDS encoding conserved core T6SS proteins are shaded in green (located in regions I and III), CDS encoding T6SS effector proteins Hcp and VrgG are colored red (located in regions II and IV, the *hcp* and *vgrG* islands), non-core CDS that are conserved among all strains are dark grey, non-conserved CDS of the T6SS that vary among strains are not colored (regions II, III and IV) and CDS flanking the T6SS are light grey. Regions of homology among strains are represented by grey shading.
