# Supplementary material for: Comparative Genomics of 12 Strains of Erwinia amylovora Identifies a Pan-Genome with a Large Conserved Core
Source: PLoS One. 2013 Feb 7;8(2):e55644. doi: 10.1371/journal.pone.0055644 (PMC3567147; doi:10.1371/journal.pone.0055644)
Supplement: Figure S3 — Comparison of the T6SS-3 loci from different strains of E. amylovora . CDS encoding conserved core T6SS proteins are shaded in green (primarily conserved core regions I, III and V but there is also a core protein in region IV of CFBP 1430 and ATCC BAA-2158), CDS encoding T6SS effector proteins Hcp and VrgG are colored red (located in conserved core region I and hcp and vgrG islands regions II and IV), non-core CDS that are conserved among all strains are dark grey, non-conserved CDS of the T6SS are not colored (region IV) and CDS flanking the T6SS are light grey. Regions of conservation among strains are represented by grey shading. (PDF) [file pone.0055644.s003.pdf]

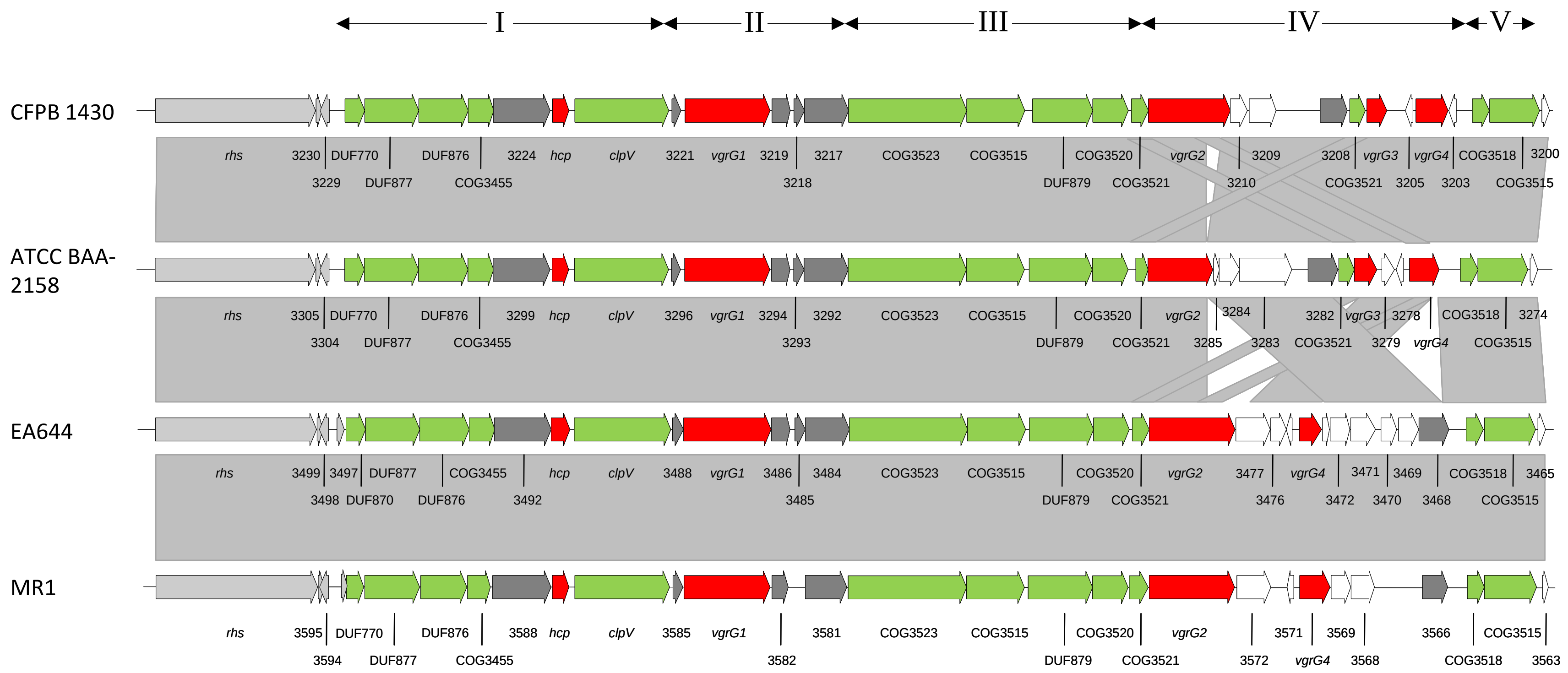

Supplementary Figure 3. Comparison of the T6SS-3 loci from different strains of *E. amylovora*. CDS encoding conserved core T6SS proteins are shaded in green (primarily conserved core regions I, III and V but there is also a core protein in region IV of CFBP 1430 and ATCC BAA-2158), CDS encoding T6SS effector proteins Hcp and VrgG are colored red (located in conserved core region I and *hcp* and *vgrG* islands regions II and IV), non-core CDS that are conserved among all strains are dark grey, non-conserved CDS of the T6SS are not colored (region IV) and CDS flanking the T6SS are light grey. Regions of conservation among strains are represented by grey shading.
