# Supplementary material for: Comparative Genomics of 12 Strains of Erwinia amylovora Identifies a Pan-Genome with a Large Conserved Core
Source: PLoS One. 2013 Feb 7;8(2):e55644. doi: 10.1371/journal.pone.0055644 (PMC3567147; doi:10.1371/journal.pone.0055644)
Supplement: Figure S4 — Comparison of plasmid pEA30 of CFBP 2585 (Ea495) to the RA3 plasmid of Aeromonas hydrophila . The RA3 plasmid is the archetype of the IncU plasmids which are a distinct group of mobile elements with highly conserved backbones and variable antibiotic resistance gene cassettes. Conservation between pEA30 and RA3 (represented by the grey shaded lines) is limited to the conserved backbone of replication, maintenance and transfer related genes. (PDF) [file pone.0055644.s004.pdf]

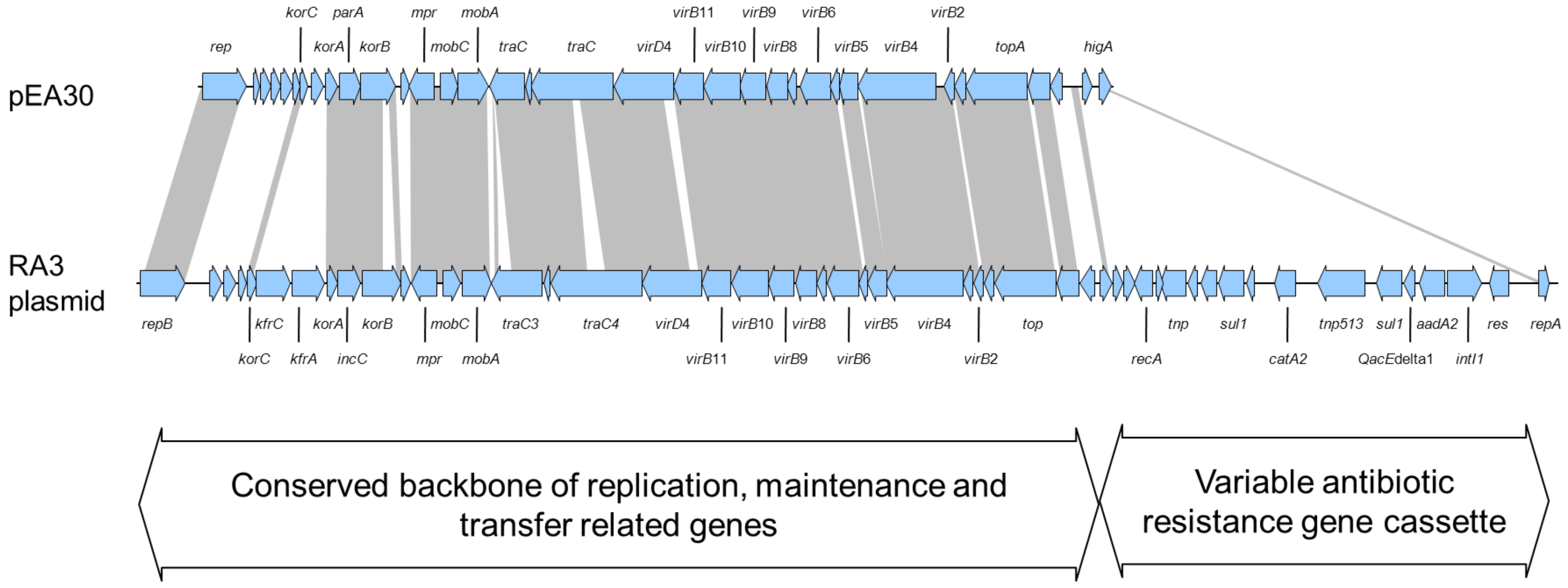

Supplementary Figure 4. Comparison of plasmid pEA30 of CFBP 2585 (Ea495) to the RA3 plasmid of *Aeromonas hydrophila*. The RA3 plasmid is the archetype of the IncU plasmids which are a distinct group of mobile elements with highly conserved backbones and variable antibiotic resistance gene cassettes. Conservation between pEA30 and RA3 (represented by the grey shaded lines) is limited to the conserved backbone of replication, maintenance and transfer related genes.
