# Supplementary material for: Comparative Genomics of 12 Strains of Erwinia amylovora Identifies a Pan-Genome with a Large Conserved Core
Source: PLoS One. 2013 Feb 7;8(2):e55644. doi: 10.1371/journal.pone.0055644 (PMC3567147; doi:10.1371/journal.pone.0055644)
Supplement: Table S1 — Pan-genome loci of the E. amylovora pan-genome that contain genomic islands. When two lines are present for a pan-genome locus, two different genomic islands are present. (PDF) [file pone.0055644.s005.pdf]

Supplementary Table 1. Pan-genome loci of the *E. amylovora* pan-genome that contain genomic islands. When two lines are present for a pan-genome locus, two different genomic islands are present.

Pan-genome Locus (PL)	1430 CDS	ATCC BAA-2158 CDS	Ea644 CDS (BN439)	MR1 CDS (BN440)	Length (kb)	Summary
1	0058-0067	-	-	-	13	Markers of mobility and hypothetical proteins.
	-	-	63-65	79-86	2.4	Hypothetical proteins shared but MR1 has extra sequence-encoding markers of mobility.
3	0383 -0403	0379-0400	-	-	23.4	Markers of mobility, hypothetical proteins and DNA degradation (Dnd) host-specific restriction modification system (DndBCDFG&H) associated with S-modification of DNA instead of methylation to protect homologous DNA.
	-	-	405-434	439-466	34.1	Markers of mobility, hypothetical proteins, putative restriction modification system proteins of Type I restriction-modification system (HsdM, HsdS & HsdR)
4	560-575	558-606	767-834	828-890	18 - 51	Remnant ICE inserted near PAI-1 which is only consistent within Spiraeoideae-infecting strains. Mosaic in all Rubus-infecting strains. Major genome reduction has been undertaken in Spiraeoideae-infecting strains.
5	0671-0679	3825, 3817 & 3810	933-941	990-1007	5.6 - 17.6	Mosaic region containing multiple predicted proteins with toxin SymE, type I toxin-antitoxin system coding domains (in all strains). There are mobility markers in MR1 (and some in Ea644), but the region appears to have undergone genome reduction and rearrangement in Spiraeoideae-infecting strains. CDS matches in ATCC BAA-2158 are in smaller unaligned contigs.
6	-	795-800	1034-1045	1099-1111	4.3 - 10.4	Mosaic in Rubus-infecting strains containing Mobility markers and hypothetical proteins.
7	-	-	1357-1375	-	18	Genomic island only present in Ea644 that encodes markers of mobility, hypothetical proteins and T6SS effector protein hcp1.
9	-	-	1887-1920	-	19.9	Mobility markers and hypothetical proteins.
12	1828-1837	1883-1888	-	-	7.8	Mobility markers and hypothetical proteins. (ATCC BAA-2158 missing 4 hypothetical proteins)
18	-	-	2913-2925	3003-3018	12	Mobility markers and hypothetical proteins.
19	2558-2566	-	-	-	5.3	Mobility markers and hypothetical proteins.
	-	-	516-520	549-552	1.5	Mobility marker and hypothetical proteins
20	-	2885-2893	599-608	634-645	20.4	Mobility markers, polyketide synthases, peptide synthase and transporter. Corresponding region in Spiraeoideae-infecting strains contains CRISPR region 1 and the remnants of this island.
24	3310-3321	3388-3403	1892-1900	-	13	The Spiraeoideae-infecting strains encode mobility markers and numerous phage related proteins. Some cargo genes in this genomic island are also shared by genomic island R18 of Ea644 but the rest of the genomic island appears to be different.
