# Supplementary material for: Comparative Genomics of 12 Strains of Erwinia amylovora Identifies a Pan-Genome with a Large Conserved Core
Source: PLoS One. 2013 Feb 7;8(2):e55644. doi: 10.1371/journal.pone.0055644 (PMC3567147; doi:10.1371/journal.pone.0055644)
Supplement: Table S2 — Variable regions of interest in the pan-genome of E. amylovora. When two lines are present for a pan-genome locus, two different genomic islands are present. (PDF) [file pone.0055644.s006.pdf]

Supplementary Table 2. Variable regions of interest in the pan-genome of *E. amylovora.* When two lines are present for a pan-genome locus, two different genomic islands are present.

Pan-genome Locus (PL)	1430 CDS (EAMY_)	ATCC BAA-2158 CDS (EAIL5_)	Ea644 CDS (BN439_)	MR1 CDS (BN440_)	Length (kb)	Summary
2	0089-0092	-	-	-	4.5	Variation in Lipopolysaccharide biosynthesis genes between Spiraeoideae-infecting and Rubus-infecting strains.
	-	0082-0084	87-89	109-112	3.2	
8	-	-	-	1652-1655	3.1	Proteins with predicted functional involvement in toxin secretion including a hypothetical protein with coding domain match (6.5e-3) to effector protein SseC
10	1725-1729	1775-1779	-	-	7.1	Predicted protein involved in the uptake of and utilization of Arabinose
11	1798-1808	1850-1861	-	-	11.3	Cluster of predicted proteins involved in carbon utilisaton including a peptidase, multiple monooxygenase domain encoding CDs, putative sugar transport protein and hypothetical proteins only present in Spiraeoideae-infecting strains and ATCC BAA-2158
13	1889-1891	1942-1946	2263	2308	0.7 - 4.2	Putative lipoprotein repeats. 3 copies in S which share >93%, 5 copies in ATCC BAA-2158 which share >89% and only one copy in both R. (% identity at aa level)
14	1947-1956	2002-2010	-	-	8.2	LysR family transcription regulator, choline dehydrogenase and hypothetical proteins
15	-	-	-	2598-2601	2	Insertion element in MR1 containing IS protein, transposase and hypothetical protein
16	2200-2209	2252-2261	-	-	8.1	Putative proteins including a predicted transcriptional regulator, aminotransferase, coenzyme F390 synthetase and hypothetical proteins.
	-	-	2573-2575	2645-2647	1.6	644 and MR1 encode an insertion element containing an integrase/transposase and hypothetical protein.
17	2328-2331	2390-2394	-	-	6.5	Putative O-acetyltransferase and hypothetical proteins.
	-	-	2702-2703	2787-2788	1.8	hypothetical proteins
21	2999	3065-3076	3236	3337-3343	5 - 10	Variation in the Rhs proteins flanking T6SS-1. Both ATCC BAA-2158 and MR1 have additional sequence with multiple putative Rhs proteins of varying length. The additional sequence in these strains has regions of similarity but is not identical.
22	3015-3018	-	3259-3261	-	1.6	Variation between T6SS-1 region II
	-	3093	-	3365-3367	1.7	
24	3203-3210	3277-3285	3468-3477	3566-3572	6.5	Rearrangement in T6SS-3 region IV
25	3525-3527	3608-3610	-	-	2.1	Proteins with sequence similarity to demethylglycine dehydrogenase, ornithine cyclodeaminase and endoribonuclease L-PSP
26	3555-3561	3640-3645	-	-	3.2	Transcriptional regulator, regulator protein and hypothetical proteins
27	-	3796-3801	-	-	3.7	hypothetical proteins including those containing CDS for phage regulatory protein, SymE toxin superfamily
	-	-	3974-3993	-	10	Mobility markers (primase, recombinase phage related proteins), predicted proteins with CD hits including RHS protein, peptide synthase and colicin-like bacteriocin.
	-	3802-3805	3994-3997	-	1.7	Hypothetical proteins including those containing CDS for phage regulatory protein, SymE toxin superfamily
	-	-	-	4100-4104	16.5	Markers of mobility and hcp1 type VI secretion effector
	-	3806-3828	-	-	5.1	Includes CDS with predicted CDS for NRPKS, Endoribonuclease system, tyrosine recombinase, primase.
