# Supplementary material for: Comparative Genomics of 12 Strains of Erwinia amylovora Identifies a Pan-Genome with a Large Conserved Core
Source: PLoS One. 2013 Feb 7;8(2):e55644. doi: 10.1371/journal.pone.0055644 (PMC3567147; doi:10.1371/journal.pone.0055644)
Supplement: Table S3 — Statistics for the draft assemblies of nine E. amylovora strains sequenced in this study. (PDF) [file pone.0055644.s007.pdf]

Supplementary Table 3. Statistics for the draft assemblies of nine *E. amylovora* strains sequenced in this study.

	**Ea356**	**Ea266**	**CFBP 2585**	**01SFR-BO**	**ACW56400**	**CFBP 1232^T**	**UPN527**	**Ea644**	**MR1**
Sequencing method	454	454	454	Illumina	Illumina	Illumina	Illumina	454	454
Total # reads	227647	161110	200044	3213312	5870134	2832918	6305328	159797	143778
Total reads aligned	223743	162603	196814	2811106	5455095	2309387	5640857	150665	137282
Average read length	267	282	360	31	30	31	31	337	394
Total contigs in assembly	15	38	13	12	25	43	18	41	35
Total assembly size	3792206	3786924	3821704	3795815	3860985	3795527	3766971	3832327	3817311
Chromosome size	3763948	3758663	3767556	3767556	3766903	3767276	3766971	3803638	3789707
Chromosome coverage	15.6	12.0	18.3	22.8	41.7	18.5	46.4	12.9	13.8
pEA29 size	28258	28261	28258	28259	28251	28251	-	28689	27604
pEA29 coverage	42.5	23.9	40.6	38.5	117.4	73.1	-	54.0	59.5
pEA29 copy number	2.7	2.0	2.2	1.7	2.8	4.0	-	4.2	4.3
pEI70 size	-	-	-	-	65831	-	-	-	-
pEI70 coverage	-	-	-	-	46.6	-	-	-	-
pEI70 copy number	-	-	-	-	1.1	-	-	-	-
pEA30 size	-	-	29586	-	-	-	-	-	-
pEA30 coverage	-	-	25.5	-	-	-	-	-	-
pEA30 copy number	-	-	1.4	-	-	-	-	-	-
