# Supplementary material for: Comparative Genomics of 12 Strains of Erwinia amylovora Identifies a Pan-Genome with a Large Conserved Core
Source: PLoS One. 2013 Feb 7;8(2):e55644. doi: 10.1371/journal.pone.0055644 (PMC3567147; doi:10.1371/journal.pone.0055644)
Supplement: Text S1 — Additional text describing differences in lipopolysaccharides and type VI secretion systems among the E. amylovora genomes. (PDF) [file pone.0055644.s008.pdf]

Supplementary document belonging to:

Comparative genomics of 12 strains of *Erwinia amylovora* identifies a pan-genome with a large conserved core

R.A. Mann, T.H.M. Smits, A. Bühlmann, J. Blom, A. Goesmann, J.E. Frey, K.M. Plummer, S.V. Beer, J. Luck, B. Duffy and B. Rodoni

Supplementary text:

Lipopolysaccharides

Lipopolysaccharides (LPS) are a major component of the cell surface of gram-negative bacteria and are typically thought to be a protective barrier against host defense molecules. Mutational analysis of a LPS biosynthesis gene (*waaL*) in Spiraeoideae-infecting strains of *E. amylovora* has shown LPS to be involved in virulence [1]. Comparison of the LPS biosynthetic gene cluster of *E. amylovora* has identified a multiple-gene substitution between Spiraeoideae-infecting and *Rubus*-infecting strains (CFBP 1430, EAMY_0089-0092 vs. ATCC BAA-2158, EAIL5_0082-0084) [2]. In Spiraeoideae-infecting strains, the core region of the LPS biosynthetic gene cluster contains genes encoding three glycosyltransferases and an LPS ligase (Spiraeoideae-type *waaL*), whereas only two glycosyltransferases and a different LPS ligase (*Rubus*-type *waaL*) are located in the same region in *Rubus*-infecting strains. These coding domains share little to no similarity at the amino acid level between *Rubus*- and Spiraeoideae–infecting strains and this genotypic difference was confirmed by PCR analysis of the associated DNA region in 31 *Rubus*- and Spiraeoideae-infecting strains [2].

Type VI secretion systems

The type VI secretion system (T6SS) has been identified in at least a quarter of the sequenced Gram-negative bacteria, with varying roles in virulence, host-specificity, symbiosis and interbacterial interactions [3]. Three type VI secretion system gene clusters have been identified in *E. amylovora* [4] but their exact role in this species is unknown. Pan-genome

comparisons have allowed identification of variation among the *E. amylovora* strains in the T6SS clusters 1 and 3. Comparison of these T6SS clusters among closely related *Erwinia* and *Pantoea* species previously identified conserved core regions and variable *hcp* and *vgrG* islands [5]. In this study, variation between strains of *E. amylovora* was primarily found within the non-conserved *hcp* and *vgrG* islands of T6SS-1 regions II and IV and T6SS-3 region IV (**Supplementary Figures 2 and 3**).

The only two differences observed in the conserved core regions of the three T6SSs of *E. amylovora* are within the region III of T6SS-1. There is a frame shift in *clpV* of MR1 causing the translated product to be truncated at amino acid 196 and the *Rubus*-infecting strains Ea644 and MR1 each contain additional sequence (approximately 1300 bp sharing 99% identity) between COG3520 and *clpV* (though the annotation between these strains varies slightly) (**Supplementary Figure 2**). Comparison of CDS BN439_3246- 3249 in Ea644 encoded in the additional sequence (corresponding to BN440_ 3354 and 3355 in MR1), to proteins in GenBank using the BLASTp algorithm revealed sequence identity (52 – 65% aa identity) to CDS in the corresponding loci in the T6SS-1 of *E. pyrifoliae* DSM 12163 (EPYR_00667 and EPYR_00668) [5,6].

The *hcp* and *vgrG* island in T6SS-1 region II is identical between the Spiraeoideae-infecting strains and *Rubus*-infecting strain Ea644. In both ATCC BAA-2158 and MR1 there is an additional non-coding sequence (approximately 1500 bp) between COG3517 and *hcp* that shares 99% nucleotide identity (**Supplementary Figure 2**). Comparison of this region to known proteins in GenBank using the BLASTx algorithm showed similarity to T6SS hypothetical protein, PSL1_3090 (64% aa identity, 99% coverage), of *Pantoea* sp SL1_M5 [7]. In the sequence following *hcp* in ATCC BAA-2158 orthologs of the hypothetical proteins of CFBP1430 EAMY_3014 to EAMY_3016 are missing. This region in MR1 lacks the hypothetical proteins EAMY_3015 to EAMY_3018, but encodes three CDS encoding predicted hypothetical proteins in its place (BN440_3364 – BN440_draft2_3367) (**Supplementary Figure 2**). Two of these CDS, BN440_3366 and BN440_3367 share 93% and 96% amino acid identity to EPYR_00656, a hypothetical protein located in the corresponding region of the *E. pyrifoliae* DSM 12163 T6SS-1 gene cluster [5,6].

In T6SS-1 region IV, there is a variable region between CFBP1430 CDS EAMY_3001 and EAMY_3002 (approximately 800 bp) and the *Rubus*-infecting strains (approximately 1350 bp).

Within the T6SS-3 loci, the non-conserved gene cluster designated as region IV [5] is conserved between the Spiraeoideae-infecting strains and ATCC BAA-2158, but there is variation between these strains and the *Rubus*-infecting strains MR1 and Ea644 due to a rearrangement in the region spanning between *vgr1* and COG3518 (**Supplementary Figure 3**). This rearrangement has primarily affected the length and synteny of CDS in the region (some frame shifts have also occurred) with orthologs of all predicted CDS present in at least one copy in all strains. In region IV of the Spiraeoideae-infecting strains and ATCC BAA-2158 there is an additional copy of COG3521 and a partial Vgr domain encoding CDS (**Supplementary Figure 3**).

Proteins of the Hcp and VgrG families are effector proteins of the T6SS which, in *Vibrio cholera*, form part of the secretion apparatus and are also secreted into the host cell [8]. In some bacterial species genes encoding Hcp and VgrG proteins have been located in loci separate from T6SS gene clusters [9]. In *Rubus*-infecting strain Ea644, an additional *hcp1* CDS (BN439_1367) is located on a genomic island outside of the T6SS clusters. MR1 also encodes an additional *hcp1* (BN440_4103) which shares 81% amino acid identity to BN439_1367. When compared to predicted proteins in the GenBank database both additional Hcp1 encoding CDS for Ea644 and MR1 share highest amino acid identity to PanABDRAFT_3416, a type VI secretion system effector Hcp1 from *Pantoea* sp. aB (80% and 93% respectively). As mentioned previously, ATCC BAA-2158 also contains an additional *hcp* CDS in the remnant ICE in PAI-1. This singleton Hcp has a higher amino acid identity (88%) to Hcp (PAGR_g3636) of *Pantoea ananatis* PA13 than it does to any of the Hcp encoding CDS of other *E. amylovora* strains.

Based on T6SS comparisons between different bacterial species, it has been theorized that the *hcp* and *vgrG* islands are ‘hot spots’ for rearrangement [5]. Additionally, these islands are thought to play a role in acquisition of T6SS effectors and the development of novel, evolved VgrG and Hcp proteins among *Erwinia* and *Pantoea* species [5]. Variation within T6SS-1 and T6SS-3 identified between strains of *E. amylovora* in this study are primarily in *hcp* and *vgrG* islands. Furthermore, these variable regions shared high sequence similarity to closely related bacteria. The identification of intra-species diversity in the *hcp* and *vgrG* islands of *E. amylovora*, confirm that these regions are hot-spots for rearrangement and are likely to play an important role in the evolution and functional diversification of T6SSs [5].

References

1. Berry MC, McGhee GC, Zhao Y, Sundin GW (2009) Effect of a *waaL* mutation on lipopolysaccharide composition, oxidative stress survival, and virulence in *Erwinia amylovora*. FEMS Microbiol Lett 291: 80-87.
2. Rezzonico F, Braun-Kiewnick A, Mann RA, Goesmann A, Rodoni B, Duffy B, Smits THM (2012) Lipopolysaccharide biosynthesis genes discriminate between *Rubus*- and Spiraeoideae-infective genotypes of *Erwinia amylovora*. Mol Plant Pathol 13: 975-984.
3. Records AR (2011) The type VI secretion system: a multi-purpose delivery system with a phage-like machinery. Mol Plant-Microbe Interact 24: 751-757.
4. Smits THM, Rezzonico F, Kamber T, Blom J, Goesmann A, Frey JE, Duffy B (2010) Complete genome sequence of the fire blight pathogen *Erwinia amylovora* CFBP 1430 and comparison to other *Erwinia* spp. Mol Plant-Microbe Interact 23: 384-393.
5. De Maayer P, Venter SN, Kamber T, Duffy B, Coutinho TA, Smits THM (2011) Comparative genomics of the type VI secretion systems of *Pantoea* and *Erwinia* species reveals the presence of putative effector islands that may be translocated by the VgrG and Hcp proteins. BMC Genomics 12: 576.
6. Smits THM, Jaenicke S, Rezzonico F, Kamber T, Goesmann A, Frey JE, Duffy B (2010) Complete genome sequence of the fire blight pathogen *Erwinia pyrifoliae* DSM 12163^{T} and comparative genomic insights into plant pathogenicity. BMC Genomics 11: 2.
7. Adams AS, Jordan MS, Adams SM, Suen G, Goodwin LA, Davenport KW, Currie CR, Raffa KF (2011) Cellulose-degrading bacteria associated with the invasive woodwasp *Sirex noctilio*. ISME J 5: 1323-1331.
8. Basler M, Pilhofer M, Henderson GP, Jensen GJ, Mekalanos JJ (2012) Type VI secretion requires a dynamic contractile phage tail-like structure. Nature 483: 182-186.
9. Sarris PF, Skandalis N, Kokkinidis M, Panopoulos NJ (2010) In silico analysis reveals multiple putative type VI secretion systems and effector proteins in *Pseudomonas syringae* pathovars. Mol Plant Pathol 11: 795-804.
